# Brain-based graph-theoretical predictive modeling to map the trajectory of transdiagnostic symptoms of anhedonia, impulsivity, and hypomania from the human functional connectome

**DOI:** 10.21203/rs.3.rs-3168186/v1

**Published:** 2023-09-28

**Authors:** Diego Pizzagalli, Alexis Whitton, Michael Treadway, Ashleigh Rutherford, Poornima Kumar, Manon Ironside, Roselinde Kaiser, Boyu Ren, Rotem Dan

**Affiliations:** Harvard Medical School/McLean Hospital; Black Dog Institute, University of New South Wales, Sydney; Emory University; McLean Hospital; McLean Hospital; McLean Hospital / Harvard Medical School; McLean Hospital / Harvard Medical School; McLean Hospital / Harvard Medical School; McLean Hospital / Harvard Medical School

## Abstract

Clinical assessments often fail to discriminate between unipolar and bipolar depression and identify individuals who will develop future (hypo)manic episodes. To address this challenge, we developed a brain-based graph-theoretical predictive model (GPM) to prospectively map symptoms of anhedonia, impulsivity, and (hypo)mania. Individuals seeking treatment for mood disorders (n = 80) underwent an fMRI scan, including (i) resting-state and (ii) a reinforcement-learning (RL) task. Symptoms were assessed at baseline as well as at 3- and 6-month follow-ups. A whole-brain functional connectome was computed for each fMRI task, and the GPM was applied for symptom prediction using cross-validation. Prediction performance was evaluated by comparing the GPM’s mean square error (MSE) to that of a corresponding null model. In addition, the GPM was compared to the connectome-based predictive modeling (CPM). Cross-sectionally, the GPM predicted anhedonia from the global efficiency (a graph theory metric that quantifies information transfer across the connectome) during the RL task, and impulsivity from the centrality (a metric that captures the importance of a region for information spread) of the left anterior cingulate cortex during resting-state. At 6-month follow-up, the GPM predicted (hypo)manic symptoms from the local efficiency of the left nucleus accumbens during the RL task and anhedonia from the centrality of the left caudate during resting-state. Notably, the GPM outperformed the CPM, and GPM derived from individuals with unipolar disorders predicted anhedonia and impulsivity symptoms for individuals with bipolar disorders, highlighting transdiagnostic generalization. Taken together, across DSM mood diagnoses, efficiency and centrality of the reward circuit predicted symptoms of anhedonia, impulsivity, and (hypo)mania, cross-sectionally and prospectively. The GPM is an innovative modeling approach that may ultimately inform clinical prediction at the individual level.

ClinicalTrials.gov identifier: NCT01976975

## Introduction

A major challenge in the treatment of mood disorders is to distinguish between unipolar and bipolar depression and, specifically, to predict future bipolar symptoms. Current assessments often fail to recognize the risk of developing manic symptoms in individuals seeking treatment during a depressive episode [1, 2]. It has been documented that up to 25% of individuals with major depressive disorder (MDD) might actually have an undiagnosed bipolar disorder (BD) [3], with rates reaching 50% in treatment-resistant depression [4]. This misdiagnosis can be dangerous, as standard pharmacotherapy (i.e., SSRIs) for unipolar depression can trigger or exacerbate manic symptoms [5]. Given that manic episodes can result in devastating financial, legal, and professional consequences as well as poor prognosis [1], there is a crucial need to identify pathophysiological mechanisms that predict the development of bipolar symptoms.

Predictive modeling, i.e., data-driven machine-learning models, can provide a powerful approach for predicting clinical symptoms at the individual level. The major advantage of predictive modeling over standard correlational/regression analyses is that they utilize cross-validation. In a cross-validation framework, the model is built on a subsample of the data (training set), and prediction is done on a separate subsample (testing set). This method is crucial for increasing the generalizability of the findings and reducing overfitting [6]. For psychiatric research especially, the generalization of the results to new unseen patients is vital for translating findings into clinical practice.

The connectome-based predictive modeling (CPM) [7] is a cross-validation model that maps phenotype measures (e.g., behavior, cognition) to whole-brain patterns of functional connections (hereafter “functional connectomes”). The CPM utilizes a unique method for feature selection, where the functional connections that are most strongly correlated with the phenotype are summed together to one brain metric. The CPM has been primarily used in healthy populations [8–10] but has been increasingly shown to successfully model or predict clinical conditions, including autism [11], childhood aggression [12], and cocaine abstinence [13].

Here, we propose a new brain-based predictive modeling approach and apply it to prospectively predict the trajectory of reward-related symptoms of anhedonia, impulsivity, and (hypo)mania among treatment-seeking patients with mood disorders. By employing graph theory to define brain predictors, our brain-based graph-theoretical predictive modeling (GPM) critically extends the CPM. Graph theory is a mathematical framework that enables characterization of complex networks (here, the organization of the brain) by quantifying both integrative processes and local specialization of communities (here, of brain regions) [14]. Notably, graph-theoretical metrics have been shown to track key clinical features of mood disorders, including depression severity [15, 16], rumination [17], suicidal ideation [18], and treatment response [19, 20]. Furthermore, graph-theoretical metrics have been found to differentiate between depressed individuals with BD and MDD [21, 22]. However, to date, most prior research adopted a classification approach and did not attempt to predict symptom development in individuals.

The rationale for developing the GPM comes from the observation that complex network measures, which are derived using theoretically-based mathematical definitions, inherently encompass summations of functional connections [23]. For example, global efficiency is a fundamental graph-theoretical metric that captures the level of integration across the brain network and is defined as the average inverse shortest path length. It is calculated as: $\text{Globalefficency}=\frac{1}{n}\Sigma_{i\in N}\frac{\Sigma_{j\in Nj\neq i}d_{ij}^{-1}}{n-1}$, where N is the set of nodes (brain regions) in the network, n is the number of nodes, and *d*_*ij*_ is the distance (shortest path length) between nodes i and j. As shown, it is based on a summation of distances between regions. Thus, by using graph-theoretical metrics as brain predictors, the feature selection step of the CPM (which sums connections) can be replaced with a theoretically driven and biologically meaningful summation of functional connections. Importantly, the specific graph-theoretical metric which is identified to have predictive utility from a diverse set of possible measures can further inform the type of brain network dysfunction that is associated with a given symptom.

Our study goals included the following. First, using the GPM, we aimed to predict reward-related symptoms in individuals with mood disorders, cross-sectionally and prospectively, from baseline neuroimaging functional connectomes. Our *a priori* focus was on transdiagnostic symptoms of anhedonia, impulsivity, and (hypo)mania. Notably, most neuroimaging predictive studies are cross-sectional, namely, symptoms and neuroimaging data are both collected at baseline. However, to have clinical utility, it is essential for a model to be able to predict the development of future symptoms (while accounting for baseline symptoms). To address this gap, a longitudinal clinical evaluation of symptoms was conducted.

Second, in light of the difficulty to separate between unipolar and bipolar depression, we utilized a Research Domain Criteria (RDoC) [24] approach. In the RDoC framework, psychiatric illness is conceptualized as a continuum across behavioral, psychological, and biological measurements, and allows a dimensional analysis instead of a categorical one. Thus, diagnoses based on the Diagnostic and Statistical Manual of Mental Disorders (DSM) were not considered in our prediction models and treatment-seeking individuals were enrolled based on their performance on a reward-learning task, to allow high variability in reward-related phenotypes. Third, we aimed to examine the influence of the brain state on symptom prediction. Since it has been suggested that functional connectomes acquired during tasks can enhance prediction performance [25], we hypothesized that a reinforcement-learning (RL) task would increase the prediction success of reward-related symptoms. We further hypothesized that network measures of the reward circuit would better predict reward-related symptoms compared to whole-brain global measures. Last, we formally compared the prediction performance of the GPM and the CPM.

## Materials and Methods

### Participants

A transdiagnostic sample of 80 individuals with mood disorders was recruited. The sample included 58 individuals with unipolar mood pathology (MDD, dysthymia, MDD in partial remission) and 22 individuals with bipolar mood pathology (BD type I or II, depressed, mixed, or hypomanic). Participants were treatment-seeking, although none were acutely manic or suicidal. A demographically matched sample of 32 healthy controls was also recruited to this study but were not included in analyses in light of the study’s goal of modeling and predicting symptoms among treatment-seeking patients. Participants were enrolled according to their reward-learning performance as characterized by the probabilistic reward task [26], such that each quantile of the normative distribution of reward-learning was equally represented (see [27] for details). Clinical diagnoses and eligibility were evaluated using the Structured Clinical Interview for DSM-IV [28] (Supplemental Methods). Stable antidepressants or mood stabilizing medication were allowed (see below for details about how medication was accounted for). The study was approved by the Partners Human Research Committee and participants provided written informed consent.

### Study design and evaluation of reward-related symptoms

Participants underwent an fMRI scan, including (i) resting-state and (ii) an RL task [29]. The RL dataset from this sample was previously presented [27, 30], however, resting-state datasets were not analyzed or published before. Symptoms of anhedonia, impulsivity, and (hypo)mania were measured at baseline in a separate visit before the fMRI scan and at 3- and 6-month follow-up visits. Anhedonia was assessed using the Anhedonic Depression subscale of the 62-item Mood and Anxiety Symptom Questionnaire (MASQ-AD) [31], impulsivity was assessed using the Barratt Impulsiveness Scale (BIS) [32], and (hypo)mania was assessed using the Mania subscale of the Bipolar Inventory of Symptoms Scale (BISS-mania) [33].

### MRI data acquisition and preprocessing

MRI data were collected at McLean Imaging Center on a 3T Siemens Tim Trio using a 32-channel head coil. Preprocessing of fMRI data was done using fMRIPrep [34] and CONN [35] (Supplemental Methods).

### Whole-brain functional connectomes

A whole-brain functional connectome was computed for each individual and paradigm (RL task, rest). Note that the three runs of the RL task were conjugated to compute one connectome. The cortex was parcellated using the Schaefer 200-node atlas [36]. The subcortex was parcellated using subcortical nodes from the Harvard-Oxford (HO) atlas [37]. Pearson’s correlations were computed between the average BOLD time series from all pairs of nodes and Fisher’s transforms were applied.

### Graph-theoretical analysis

Graph theoretical analyses were carried out on the weighted positive functional connectome matrices using the Brain Connectivity Toolbox [23]. Negative functional connections were not included since most graph measures can be calculated only for positive weights. For each individual and state, global measures were computed across the entire network, in addition to local measures which were calculated for each of the reward circuit’s regions: anterior cingulate cortex (ACC), caudate, putamen, nucleus accumbens (NAc), lateral and medial orbitofrontal cortex (OFC) (see code availability section). (i) *Global measures:* characteristic path length, global efficiency, mean clustering coefficient, mean local efficiency, mean betweenness centrality. (ii) *Local measures:* clustering coefficient, local efficiency, betweenness centrality.

The above graph theoretical measures can be grouped into measures of integration, segregation, and centrality [23]. (i) *Integration:* the ability to rapidly combine information across remote brain regions. It includes the characteristic path length which is the average shortest path length between all pairs of nodes and global efficiency which is the average inverse shortest path length. (ii) *Segregation:* quantifies the presence of densely interconnected brain regions and includes the clustering coefficient and local efficiency. The clustering coefficient is the fraction of a node’s neighbors (nodes that are directly connected to the node) that are also neighbors of each other. The local efficiency is the efficiency of the subgraph of a node that contains only its neighbors. (iii) *Centrality:* the importance of a region for efficient communication. It includes betweenness centrality, which is the fraction of the shortest paths that pass through a node.

### Brain-based graph-theoretical predictive modeling (GPM)

**Figure 1:**
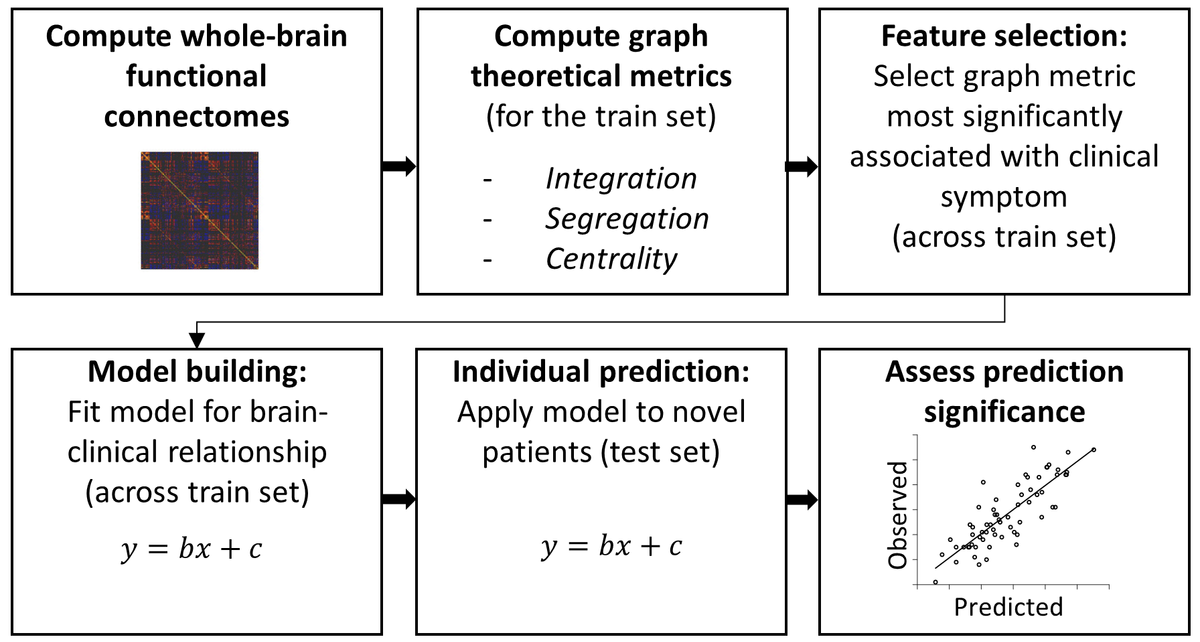
Illustration of the brain-based graph-theoretical predictive modeling (GPM). The GPM utilizes cross-validation and includes the following steps: (i) calculation of whole-brain functional connectomes for each individual; (ii) computation of graph-theoretical metrics from brain connectomes; (iii) feature selection: choosing the graph metric most strongly associated with the clinical symptom; (iv) model building: mapping between graph metric and clinical symptom; (v) model prediction: applying the model to previously unseen data. The model is built on the training set (steps i-iv) and prediction is done on the testing set (step v).

The GPM is illustrated in Fig. 1 and includes the following steps for symptom prediction: (i) calculation of whole-brain functional connectomes; (ii) computation of graph-theoretical metrics from functional connectomes (measures of integration, segregation, centrality); (iii) feature selection: choosing the graph metric (only one) which is most strongly associated with the clinical symptom. Note that this can be either a global metric, e.g., global efficiency, or a metric of a specific region, e.g., centrality of the left caudate; (iv) model building: mapping between graph metric and clinical symptom; (v) model prediction: applying the model to previously unseen data. The GPM utilized leave-one-out cross-validation (LOOCV). In LOOCV, the data are divided in each iteration into a training set (N-1 subjects, where N is the sample size) and a testing set (remaining subject). The model is built on the training set (steps i-iv) and prediction is done on the testing set (step v). This procedure is repeated N times to obtain a predicted score for all participants. Note that LOOCV was chosen due to the modest sample size, and similar results were obtained with 10-fold cross-validation (see Supplemental Results).

Model building was accomplished using multiple regression while controlling for all other baseline symptoms and psychotropic medication load [38] (see Supplement for a list of medications). For example, when predicting anhedonia at baseline, (hypo)mania and impulsivity at baseline were covaried. For predicting symptom severity at follow-up, symptom severity (for all symptom scales) at baseline was covaried. These covariates were not included in the feature selection step, but rather in the model building step (i.e., in the predictive model). For the prediction of (hypo)manic symptoms, due to the skewed distribution of scores, a log (x +1) transform was used where x refers to (hypo)mania scores (BISS-mania). Importantly, DSM categories (MDD, BP) were not considered in the model.

The model’s predictive performance (the correspondence between predicted and observed scores) was evaluated by the mean squared error (MSE). The MSE is defined as the average sum of the squared difference between the observed and predicted values and measures the variance of the residuals, with a smaller MSE indicating a better model. To quantify the contribution of the graph-theoretical brain predictor beyond that of baseline symptoms, we compared the GPM to a corresponding LOOCV null model. The null model was defined as the same model without the graph-theoretical brain predictor, including only baseline symptoms and psychotropic medication load. To formally compare between the GPM and null models, the corrected repeated k-fold cv test was used [39]. In addition, the relative difference in the MSE between the models was derived as follows:

MSE_diff_ = (MSE_null_ — MSE_GPM_)/MSE_null_. The same procedure was used to evaluate the prediction accuracy of the CPM. Last, since correlation is the most commonly reported metric of prediction performance in fMRI literature, we computed Pearson’s correlation between predicted and observed scores. The statistical significance of correlation was assessed using permutation testing, i.e., the observed scores were randomly shuffled between participants, and the prediction process was repeated 10,000 times to generate a null distribution.

The feature-selection step of the GPM implements a “winner-take-all” approach, i.e., only the best graph-theoretical metric is chosen to be included in the predictive model. As an alternative approach, we tested the inclusion of several graph-theoretical metrics by utilizing elastic-net algorithm to generate the predictive model. Elastic-net is a hybrid of ridge regression and lasso regularization and can be used to select the important predictors among a large set [40]. Elastic-net was implemented using *Gimlet* package for MATLAB (http://hastie.su.domains/glmnet_matlab/) [41]. Hyper-parameters were optimized using cross-validation for all possible combinations of alpha (elastic net penalty, ranging from 0 to 1 in steps of 0.05) and lambda (controls the overall strength of penalty). The model with the alpha-lambda pair that minimized the cross-validated error was selected.

Finally, to further evaluate if predictions are truly transdiagnostic and not driven by one patient group over the other, we split the sample to unipolar and bipolar disorders groups, trained the GPM on the unipolar disorders group and tested on the bipolar disorders group. Namely, the model was built based on data from individuals with unipolar disorders, and predictions were done for individuals with bipolar disorders. Due to smaller sample sizes for follow-ups, this analysis was conducted for baseline symptoms only.

### Comparison with the CPM

As a comparison to the GPM, the CPM was also applied for symptom prediction using available code [42]. The CPM was run on the whole functional connectome, including negative weights, as originally done [7], and on the positive-only weighted connectome, as done for the GPM. A threshold of *p* = 0.01 was applied for edge selection. The predictive utility of the positive-feature set (i.e., the edges positively associated with the clinical measure) and the negative-feature set (i.e., the edges negatively associated with the clinical measure) were tested. Apart from the inherent differences between the CPM and the GPM, all other methodological choices were kept identical (see Supplemental Methods for an illustration of the differences between the models). Note that the null model was the same for the CPM and GPM, since it includes only baseline symptoms and medication load.

## Results

### Clinical characteristics and symptom trajectories

**Figure 2:**
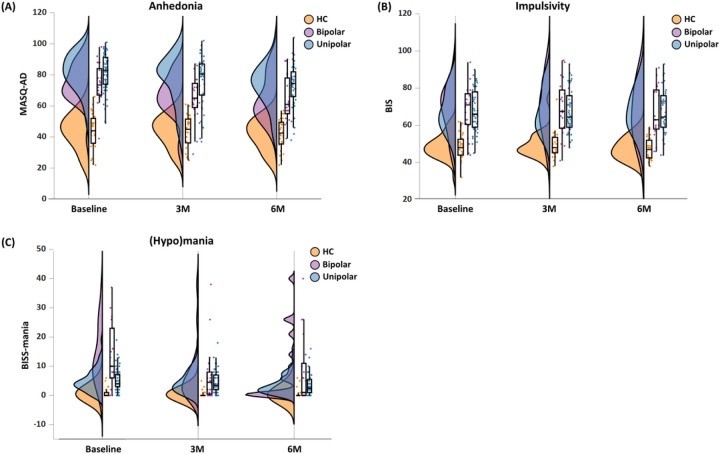
Trajectories of anhedonia, impulsivity, and (hypo)mania symptoms. The distributions (using violin plots), scatter plots, and boxplots of (**A**) anhedonia, (**B**) impulsivity, and (**C**) (hypo)mania symptoms are presented for baseline, 3-month (3M) and 6-month (6M) follow-ups. A demographically matched group of 32 healthy controls (HC) (age=28.40±7.72, 17 female) is included for comparison.

The clinical and demographic characteristics of the sample are presented in Table 1. Eight and 13 participants, respectively, were lost to follow-up at 3-month and 6-month. BIS scores at baseline were missing from 6 participants. In addition, BISS-mania scores at 3- and 6-month were missing from 1 participant; BIS and MASQ-AD scores were missing from 1 participant at 3-month follow-up and from 2 participants at 6-month follow-up. The distributions of anhedonia, impulsivity, and (hypo)mania symptoms across baseline, 3- and 6-month follow-ups are shown in Fig. 2. The list of medications used by participants (Supplemental Table 1) and the cross-correlations among symptoms over time (Supplemental Table 2) are presented in the Supplement.

### GPM symptom prediction at baseline

**Figure 3:**
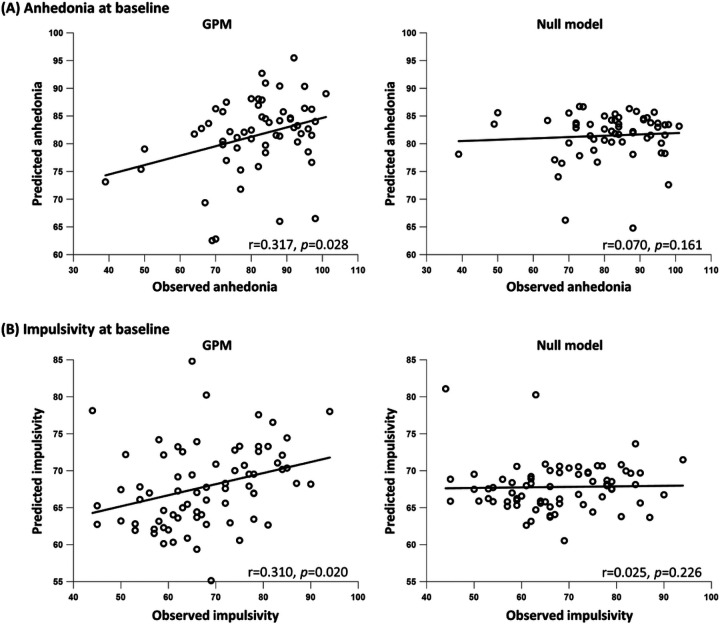
The GPM predicted anhedonia and impulsivity at baseline. Predicted clinical scores (y axis) are presented as a function of the observed clinical scores (x axis). For each symptom, the predictions of the GPM are presented at the left panel and the predictions of the corresponding null model (without the brain predictor) are presented at the right panel. (**A**)Anhedonia was predicted by the GPM from the global efficiency of the functional connectome during the reinforcement-learning task. (**B**) Impulsivity was predicted by the GPM from the centrality of the left anterior cingulate cortex during resting-state. All *p* values were computed using permutation testing. Prediction was done while controlling for other baseline symptoms and medication load.

At baseline, the GPM predicted anhedonia from the global efficiency of the functional connectome during the RL task (Fig. 3A) [N = 61; GPM: r = 0.317, *p* = 0.028 permutation testing, MSE = 152.60; null model: r = 0.070, *p* = 0.161 permutation testing, MSE = 172.15]. Decreased global efficiency was associated with greater anhedonia. The GPM outperformed the null model (t = 4.02, *p* < 10^−4^ corrected repeated k-fold CV test) and had an 11.35% lower MSE relative to the null model.

The GPM predicted impulsivity from the centrality of the left ACC during resting-state (Fig. 3B) [N = 73; GPM: r = 0.310, *p* = 0.020 permutation testing, MSE = 119.52; null model: r = 0.025, *p* = 0.226 permutation testing, MSE = 136.78]. Increased ACC centrality was associated with greater impulsivity. The GPM outperformed the null model (t = 5.03, *p* < 10^−4^ corrected repeated k-fold CV test) and had a 12.62% lower MSE relative to the null model. In a post-hoc analysis, we tested whether specific subtypes of impulsivity (as measured by the six subscales of the BIS, using a Bonferroni corrected threshold of *p* < 0.008)) could be predicted from the centrality of the left ACC. The GPM predicted the non-planning/self-control subscale of the BIS [N = 73; GPM: r = 0.406, *p* = 3·10^−4^ permutation testing, MSE =11.72; null model: r = 0.170, *p* = 0.052 permutation testing, MSE = 13.82]. The GPM outperformed the null model (t = 25.30, *p* < 10^−4^ corrected repeated k-fold CV test) and had a 15.17% lower MSE relative to the null model.

### GPM symptom prediction at 6-month follow-up

**Figure 4:**
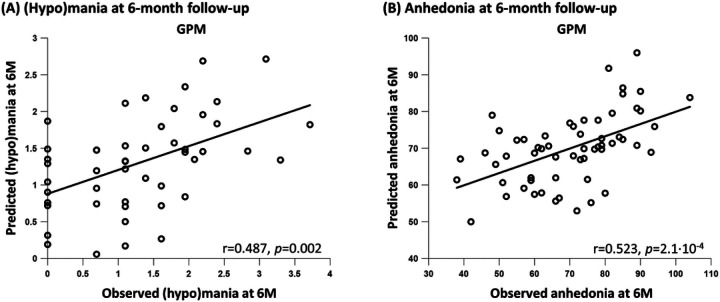
The GPM predicted (hypo)mania and anhedonia at 6-month follow-up. Predicted clinical scores (y axis) are presented as a function of the observed clinical scores (x axis). (**A**) (Hypo)mania at 6-month (6M) follow-up was predicted by the GPM from the local efficiency of the left nucleus accumbens during the reinforcement-learning task. (**B**) Anhedonia at 6-month follow-up was predicted by the GPM from the centrality of the left caudate during resting-state. All *p* values were computed using permutation testing. Prediction was done while controlling for all baseline symptoms and medication load.

At 6-month follow-up, the GPM predicted (hypo)mania from the local efficiency of the left NAc during the RL task (Fig. 4A) [N = 51; GPM: r = 0.487, *p* = 0.002 permutation testing, MSE = 0.708; null model: r = 0.400, *p* = 0.003 permutation testing, MSE = 0.785]. Increased NAc efficiency was associated with greater (hypo)mania. The GPM outperformed the null model (t = 6.36, *p* < 10^−4^ corrected repeated k-fold CV test) and had a 9.82% lower MSE relative to the null model. The GPM predicted anhedonia at 6-month follow-up from the centrality of the left caudate during resting-state (Fig. 4B) [N = 64; GPM: r = 0.523, p = 2.1·10^−4^ permutation testing, MSE = 164.23; null model: r = 0.418, *p* = 7.4·10^−4^ permutation testing, MSE = 187.16]. Increased centrality of the caudate was associated with greater anhedonia. The GPM outperformed the null model (t = 4.75, *p* < 10^−4^ corrected repeated k-fold CV test) and had a 12.25% lower MSE relative to the null model.

### GPM prediction using elastic-net

The inclusion of several graph-theoretical predictors by utilizing elastic-net did not result in an overall better predictive performance than the “winner-take-all” approach (Supplemental Results).

### GPM using data-splitting: training on unipolar disorders and predicting for bipolar disorders

The GPM predicted anhedonia at baseline from the global efficiency of the functional connectome during the RL task [train (unipolar): N = 45; test (bipolar): N = 16; GPM: MSE = 172.59; null model: MSE = 247.90]. The GPM had a 30.38% lower MSE relative to the null model. The GPM predicted impulsivity at baseline from the centrality of the right ACC during resting-state [train (unipolar): N = 53; test (bipolar): N = 20; GPM: MSE = 177.53; null model: MSE = 202.27]. The GPM had a 12.23% lower MSE relative to the null model. Notably, these results are similar to the ones obtained when running the GPM on the whole transdiagnostic sample using cross-validation.

### CPM symptom prediction

Baseline anhedonia was predicted from the CPM’s negative-feature set during the RL task [N = 61; CPM: r = 0.281, *p* = 0.020 permutation testing, MSE = 164.34; null model: r = 0.070, *p* = 0.161 permutation testing, MSE = 172.15]. The CPM outperformed the null model (t = 3.66, *p*= 1.3·10^−4^ corrected repeated k-fold CV test) with a 4.54% lower MSE relative to the null model. Compared to the GPM, the CPM had a 7.69% higher MSE suggesting decreased performance, however, the differences between the models were not significant (t = 1.19, *p* = 0.116 corrected repeated k-fold CV test). Similar results were obtained with the positive-only weighted functional connectome (Supplemental Results). The CPM did not predict any other symptoms at baseline or follow-up.

## Discussion

Here, we developed and optimized a new brain-based model – the GPM – to predict reward-related symptoms in treatment-seeking individuals with mood disorders. Within the GPM, the prediction of symptoms was based on graph-theoretical measures of brain functional organization. We found that the efficiency and centrality of the reward circuit, specifically the ACC, caudate, and NAc, predicted symptoms of anhedonia, impulsivity, and (hypo)mania, cross-sectionally and prospectively. Importantly, the prediction of mood symptoms was done in an RDoC approach which was agnostic to DSM categories.

Anhedonia at baseline was predicted by the GPM from the global efficiency of the functional connectome during an RL task. Specifically, greater anhedonia was associated with decreased global efficiency across the brain. Notably, a data-splitting analysis indicated that the prediction was cross-diagnostic: specifically, anhedonia in individuals with bipolar disorders was predicted after training the GPM on individuals with unipolar disorders. Anhedonia is one of the two cardinal symptoms of depression and is defined as a loss of pleasure, motivational drive, or lack of reactivity to pleasurable stimuli [43]. Global efficiency is a core network measure of integration and reflects the network’s ability to pass information through short paths, which are considered important for the flow of signals and communication [44]. To the best of our knowledge, this is the first demonstrated link between global efficiency or integration and anhedonia. In agreement with our results, decreased global integration was previously reported in MDD. For example, decreased global integration was demonstrated in a large sample of 821 MDD patients relative to healthy controls [45].

Anhedonia at 6-month follow-up was predicted by the GPM from the centrality of the left caudate during resting-state. Greater anhedonia was associated with increased centrality of the caudate in the functional connectome. Measures of centrality characterize the contribution of a brain region to the cohesiveness of the network [46]. There is emerging evidence associating anhedonia with increased centrality of the caudate during rest. A recent longitudinal study on a large cohort with adolescent depression found that centrality of the ventral striatum was positively associated with anhedonia at baseline, 2- and 4-year follow-ups [47]. Anhedonia can be further divided into anticipatory and consummatory components, where anticipatory anhedonia is an impairment to predict future pleasure and consummatory anhedonia is the reduced ability to experience pleasure [48]. In adolescence, anticipatory anhedonia was found to correlate with the centrality of the caudate [49]. In addition, anticipatory anhedonia was positively correlated with the functional connectivity of the ventral caudate and the middle temporal gyrus [50].

Conversely, impulsivity was predicted at baseline by the GPM from the centrality of the left ACC during resting-state. Critically, evidence of transdiagnostic generalization emerged: the GPM derived from individuals with unipolar disorders (centrality of the right ACC during resting-state) predicted baseline impulsivity among individuals with bipolar disorders. Greater impulsivity was associated with increased ACC centrality. Impulsivity is a feature of several psychiatric disorders including bipolar disorder, attention-deficit/hyperactivity disorder, and substance use disorders. Impulsivity can be defined as the tendency to act without adequate forethought or conscious judgment of potential consequences [51]. Several models have suggested that impulsivity is a heterogeneous construct composed of different dimensions. For example, the BIS divides impulsivity into three components: motor (acting without thinking), attentional (lack of focus on the task), and non-planning (orienting toward the present instead of the future).

The ACC has been suggested to play a key role in impulsivity, due to its involvement in diverse higher-order cognitive processes related to executive functioning, such as conflict monitoring, attention, task switching, and response inhibition [52]. In patients suffering from focal brain injuries, greater impulsivity was associated with lesions to the ACC [53]. Difficulties in perseverance, a subtype of impulsivity, were correlated with greater functional connectivity of the ACC with prefrontal regions [54]. Neurochemistry measures of the ACC were implicated in impulsivity, specifically serotonin [55, 56] and glutamate [57–59]. Moreover, neuroimaging studies have supported the involvement of the ACC in the non-planning impulsivity subtype [60, 61], in accordance with our findings.

(Hypo)mania at 6-month follow-up was predicted by the GPM from the local efficiency of the left NAc during RL task, with greater (hypo)mania associated with increased NAc efficiency. Mania is described as a distinct period of abnormally and persistently elevated, expansive, or irritable mood [28]. Notably, the NAc has been strongly implicated in manic symptoms. For example, transient (hypo)mania is the most commonly observed side effect after deep brain stimulation to the NAc [62–64]. Recently, preliminary evidence associated mania with increased structural and functional connectivity of the NAc. In a community sample, (hypo)mania proneness was positively correlated to structural connectivity between the NAc and the OFC and amygdala [65]. Increased functional connectivity between the NAc and the ventromedial PFC was shown in BP patients relative to healthy controls [66].

For clinical translation, it is important to identify the cognitive state (e.g., rest or RL task) that can maximize the predictive accuracy of the GPM. Contrary to our hypothesis, the RL task did not amplify the prediction success of reward-related symptoms, but rather a similar number of predictions was made from the RL task and resting-state. However, we note that data for the two states were collected using different imaging sequences: single-band for the RL vs. multiband for the resting-state. The states also differed in the scan duration: 31.5 and 6.67 minutes, respectively, for the RL task and resting-state. Despite a longer scan duration for the RL task, the number of volumes was greater for the resting-state (800 vs. 630) due to multiband acceleration. Thus, these differences in imaging parameters require future investigation to delineate more clearly the contribution of cognitive activities to the prediction accuracy of reward-related symptoms.

Most symptom predictions were based on regional network measures of the reward circuit, and not global ones, in accordance with our hypothesis. This finding resonates with numerous evidence for the pivotal role of the reward circuit in the pathophysiology of mood disorders [67] and extends previous literature by demonstrating the predictive utility of its network attributes. Importantly, different network measures were found to predict baseline symptoms and future ones. Since the ultimate goal of predictive modeling in psychiatry is to map future symptoms (and not baseline ones, which are generally known and can be assessed directly), our finding underscores the critical importance of longitudinal studies which monitor symptom trajectories. In addition, we note that the GPM outperformed the CPM for symptom prediction.

This result was found when applying the CPM on either the functional connectome including negative weights, or on the positive-only functional connectome.

Brain-based predictive modeling holds the cardinal promise to transcend the current paradigm of psychiatric assessments which are based solely on a patient’s subjective report. An accurate, reproducible, predictive model can support important clinical decision making, such as selecting among possible treatments, initiating preventive strategies, and risk monitoring. While our study took a step towards achieving that goal, clearly more large-scale neuroimaging studies in individuals with mood disorders, with greater sample sizes, are essential to establish the predictive utility of the GPM. Nevertheless, our findings highlight several brain metrics that can be further tested as potential biomarkers, particularly for identifying individuals with depression that are at risk for developing bipolar symptoms. Although taking the leap toward translational neuroscience is still rarely done, a few studies have directly investigated the clinical utility of neuroimaging biomarkers. For example, Kelley et al. assigned treatment for patients with MDD based on fluorodeoxyglucose positron emission tomography activity in the insula [68].

Several limitations should be noted. First, our sample was moderate in size and thus the findings should be carefully validated with larger and independent datasets. Second, the range of manic symptoms in our sample was relatively narrow and included mainly hypomania symptoms. Third, symptoms at 3-month follow-up were not predicted from brain measures due to high similarity with baseline scores (i.e., variance at 3 months was captured mostly by baseline scores). Moreover, impulsivity was quite stable across the three time-points. This may be due to the BIS capturing predominantly trait impulsivity. We note that a follow-up period of 6 months was chosen to facilitate the retention of participants, however, symptoms might change more substantially with longer follow-ups. Fourth, we did not test the influence of preprocessing choices or the parcellation atlas on prediction performance. Finally, alternative approaches to estimating functional connectomes may yield complementary information, e.g., aggregating fMRI data across paradigms, (task and rest) [69], but were outside the scope of this investigation and merit future study. The optimal neuroimaging setting for detecting individual differences that are clinically relevant remains an ongoing research topic and an important direction for future research in psychiatry.

## Conclusions

The GPM is an innovative tool that can map between clinical symptoms and network neuroimaging markers at the individual level. Across mood disorders, the GPM successfully predicted symptoms of anhedonic depression, impulsivity, and (hypo)mania, cross-sectionally and prospectively, from network features of the reward circuit. Ultimately, these results may have implications for prognostic indicators of mood symptoms. The clinical need is acute since a timely and effective treatment for individuals suffering from mood disorders, especially bipolar mood pathology, is crucial to reduce morbidity and mortality [70].

## Figures and Tables

**Table 1:** Clinical and demographic characteristics of the sample

	Unipolar disorders (*n*=58)	Bipolar disorders (*n*=22)
**Demographics**		
Age, years, mean ± SD (range)	28.0 ± 8.6 (18–60)	31.7 ± 13.2 (18–57)
Female, *n* (%)	41 (71.7)	12 (54.5)
Education, years, mean ± SD (range)	16.0 ± 2.8 (10–25)	16.0 ± 3.0 (10–24)
White, *n* (%)	40 (69.0)	19 (86.4)
Hispanic, *n* (%)	6 (10.3)	1 (4.5)
**Clinical diagnoses, n(%)**		
Current MDD	49 (84.5)	-
Current dysthymia	1 (1.7)	-
MDD in partial remission	8 (13.8)	-
BD-I depressed	-	7 (31.8)
BD-I mixed	-	0 (0.0)
BD-I hypomanic	-	2 (9.1)
BD-II depressed	-	9 (40.9)
BD-II mixed	-	1 (4.6)
BD-II hypomanic	-	3 (13.6)
**Medication, n(%)**		
Antidepressants	19 (32.8)	3 (13.6)
Mood stabilizers or anticonvulsants	1 (1.7)	9 (40.9)
**Reward-related symptoms mean ± SD (range)**		
Anhedonia (MASQ-AD)		
Baseline	81.5 ± 11.9 (49–101)	74.3 ± 12.9 (39–98)
3-month follow-up	76.1 ± 16.0 (37–102)	65.3 ± 14.7 (29–87)
6-month follow-up	73.1 ±14.1 (38–104)	64.4 ± 14.5 (39–90)
Impulsivity (BIS)		
Baseline	67.5 ± 10.6 (45–90)	68.0 ± 13.2 (44–94)
3-month follow-up	67.7 ± 11.5 (48–93)	69.3 ± 14.8 (41–95)
6-month follow-up	67.2 ± 11.7 (44–93)	66.9 ± 13.7 (46–91)
Mania (BISS-mania)		
Baseline	5.1 ± 3.9 (0–19)	13.6 ± 10.1 (0–37)
3-month follow-up	4.5 ± 3.8 (0–18)	6.8 ± 9.6 (0–38)
6-month follow-up	3.7 ± 3.5 (0–16)	7.9 ± 11.2 (0–40)

BD-I/II, bipolar disorder type I/II; BIS, Barratt Impulsiveness Scale; BISS-mania, Bipolar Inventory of Symptoms Scale Mania subscale; MASQ-AD, Anhedonic Depression subscale of the 62-item Mood and Anxiety Symptom Questionnaire; MDD, major depressive disorder.
